# Supplementary material for: Evidence in cortical folding patterns for prenatal predispositions to hallucinations in schizophrenia
Source: Transl Psychiatry. 2020 Nov 6;10:387. doi: 10.1038/s41398-020-01075-y (PMC7648757; doi:10.1038/s41398-020-01075-y)
Supplement: Supplementary file 1 — Supplemental Material [file 41398_2020_1075_MOESM1_ESM.docx]

**Supplementary Information**

**Supplementary Methods**

**Participant recruitment and scanning acquisition**

Participants in the UK dataset were recruited for a randomised, double blind, placebo-controlled trial evaluating the benefit of minocycline on negative symptoms of schizophrenia (BeneMin) in patients with a schizophrenia-spectrum disorder that had begun within the past 5 years ^1^. From the six local neuroimaging centres involved, three were excluded due to gender imbalances between groups, leaving N=85 patients scanned in Manchester, N=24 in Cambridge, and N=13 in Edinburgh for the comparison of brain morphology. The study was registered as an International Standard Randomised Controlled Trial, number ISRCTN49141214, and the EU Clinical Trials register (EudraCT) number is 2010-022463-35I. The North West Manchester Research Ethics Committee (reference number 11/NW/0218) approved the study. Patients with schizophrenia were recruited from the Shanghai Mental Health Centre for a study to examine striatal brain activations during monetary and affective incentive processing ^2^. Healthy age- and gender-matched controls were recruited through advertisements from local communities. The study was approved by Shanghai Mental Health Centre and the Institute of Psychology. Written, informed consent was obtained from participants for both studies.

For both datasets, symptom severity was assessed using the Positive and Negative Symptom Scale (PANSS) and high-resolution T1-weighted structural images were acquired on a 3T MRI scanner. The principle differences between the datasets were in their design. The UK study was a randomised, longitudinal trial with clinical measures taken over a 12-month period. Only the pre-randomised data were used in this study. The Shanghai dataset was cross-sectional, and recruited healthy controls.

**Hallucination grouping**

Patients were grouped into those with (H+) and without (H-) hallucinations, defined by a score of > 2 (H+) and <= 2 (H-) on the PANSS P3 item for hallucinatory behaviour at the time of assessment, as has been used previously for assessing the underlying brain structure related to hallucination presence ^3^. The P3 hallucinatory behaviour item measures severity of hallucinations on a scale from 1 (absent) to 7 (extreme) in the week before clinical assessment. Although not a measure of lifetime history of hallucinations, the P3 score shows good convergent validity with other questionnaires assessing hallucination presence ^4, 5^. The P3 score was additionally available for the UK dataset at 2, 4, 6, 9, and 12 months. The average P3 measured periodically over the 1-year period was compared to P3 scores at randomization in attempt to closer serve as a proxy for lifetime hallucination history (Table 1).

**Paracingulate sulcus manual tracing measurement and reliability analysis**

The length of the PCS was manually measured on both hemispheres of all N=183 images from the UK sample according to a previously described protocol ^6^, with open access at www.repository.cam.ac.uk/handle/1810/264520. Briefly, individual images were imported into Multi-image Analysis GUI (Mango) medical imaging visualization software (v.4.0.1, <http://ric.uthscsa.edu/mango/>) and manually linearly aligned with 6 orthogonal degrees of freedom (DOF) to the plane of the anterior and posterior commissures (ACPC). A coordinate origin set to the anterior commissure along the ACPC line defined the quadrants for PCS measurement. Manual tracing was performed on a sagittal slice, +/-4 mm from the transverse medial line, according to the hemisphere being traced. The cingulate sulcus was first defined as the first major sulcus running dorsal to the corpus callosum in the anterior–posterior direction. The PCS was then identified, if present, as a salient sulcus, running parallel, horizontal and dorsal to the cingulate sulcus, and visible for >=3 sagittal slices measured from the medial (x=0) plane. The PCS was measured using the “trace line” function in Mango, starting at the point in the first quadrant (y>0, z>0) at which the sulcus runs in a posterior direction to its end point, which could fall outside the first quadrant if the sulcus is continuous. Where the PCS was discontinuous, additional segments were included if the interruption between segments was <20 mm and if they also began in the first quadrant.

To assess the reliability of the manual PCS measurement protocol, four independent raters were trained de novo based on the cited protocol, such that each participant scan from the UK sample (n = 122) was evaluated by at least 2 raters. All raters used the same tracing program (Mango) and style (keypad). Prior to reliability testing, raters measured the PCS from 10 training scans previously labelled by JRG. Labelled training scans were reviewed by one of two experienced raters (JRG, who developed the protocol, or CPER, trained by JRG, both with over 3 years of experience with brain MRI processing and segmentation and have each measured the PCS in >100 subject scans) and discrepancies were discussed and clarified. The consistency of PCS length measurements between raters was evaluated with the intraclass correlation coefficient (ICC). Results are given in Supplementary Table S3.

**Automated sulcal segmentation for measurement of the superior temporal and paracingulate sulcus**

Initial automatic sulcal segmentation was performed using BrainVISA version 4.5 software (<http://brainvisa.info>) with standard parameters. For comparability to manual tracing, T1-weighted images were first linearly aligned to the ACPC plane and brought into MNI152 template space using a 6 DOF alignment with FSL commands. Aligned images were imported into the BrainVISA’s Morphologist 2015 pipeline. No non-linear spatial normalization was applied to MRIs to overcome potential bias that may result from the sulcus shape deformations induced by the non-linear warping process. Using standard parameters, images were corrected for spatial inhomogeneities, skull-stripped, segmented into grey matter (GM), white matter (WM), and cerebrospinal fluid (CSF), separated by hemisphere, and had their 3D surfaces reconstructed corresponding to the GM-WM and GM-CSF interface. Cortical folds were automatically detected based on the image intensity of the MRI, a stable and robust definition that is not affected by variations in cortical thickness or GM/WM contrast ^7^. BrainVISA can provide automatic reliable recognition of the majority of primary cortical sulci, including the STS ^8^, but is unable to accurately label the PCS and other secondary/tertiary sulci due to their high morphological variability. STS labels were visually inspected in Anatomist viewer. The PCS was manually labelled (CPER and MA) from the segmented folds following the same definition outlined in the manual tracing protocol. Native space summary measurements of sulcal length and depth were automatically computed for automatically delineated STS and manually labelled PCS. The consistency between PCS length measurements derived from manual and semi-automated (BrainVISA) segmentations were compared with the ICC, analogous to the ICC calculations between raters for the manual method. Results are given in Supplementary Table S4.

**Creation of group-wise average sulcal maps for the paracingulate and superior temporal sulci**

To visualize the average morphology of the sulci of interest in each group (H+, H-, HC), we created 3D maps of the PCS and STS in standard MNI152 stereotaxic space. First, graphical representations of the sulci were converted to 3D volumes using the BrainVISA ‘Create Sulcus Label Volume’ tool. For each image, the resultant volumetric PCS labels were overlaid on the bias-corrected input MRI using FSLeyes to visually inspect each label and ensure later comparability to the manual tracing. For each hemisphere of each group, we used FSL commands to merge the appropriate sulcal labels. Since individual PCS labels were thin, spanning approximately 2 voxels in width, resultant sulcal maps were systematically smoothed by iteratively increasing sigma until an optimal level was reached via visual inspection at sigma=0.75. The UK and Shanghai datasets were merged to produce average sulcal maps maps since there were no significant between-sample differences in the morphological classification of the PCS in either our sample or in prior studies ^9^. The positional difference between group sulcal maps was quantified using fslmaths. The average sulcal maps were visualized in FSLeyes for group comparisons of sulcal geometry.

**Cortical surface reconstructions**

Cortical thickness (CT) and local gyrification index (LGI) were calculated using the FreeSurfer analysis package (v.6.0, <http://surfer.nmr.mgh.harvard.edu/>). Cortical surfaces from each participant's T1-weighted MRI scan were automatically reconstructed using the recon-all pipeline. The technical procedures have been detailed in previous publications ^10-13^ and have been demonstrated to show good test-retest reliability across scanner manufacturers and field strengths ^14^. Resultant cortical surfaces were visually inspected for quality and manually corrected in FreeView, when appropriate, according to FreeSurfer user guidelines. Minor interventions were required, typically around the temporal poles due to non-cortex material included in the pial surface. There were no differences in the degree of manual intervention required between groups. Images were then re-processed, incorporating the corrections to improve reconstructed surface accuracy. Cortical thickness (CT) was automatically generated from the Freesurfer processing pipeline, and smoothed using a Gaussian kernel of FWHM of 10 mm. Local gyrification index (LGI) was calculated according to the method developed and described in detail by Schaer et al. ^15, 16^. LGI calculations were visually assessed for accuracy by overlaying LGI values over the cortical surface in TkSurfer. LGI was smoothed with a FWHM kernel of 5 mm, as previously ^6^.

**Local gyrification index as a proxy for sulcal length and depth**

Individual differences in cortical anatomy are characteristic of human brains, yet arbitrary interruptions and branching of cortical sulci renders sulcal morphology sufficiently distinct from variability in other brain morphology measures. A number of methods have been proposed for automated identification of sulci, but are generally limited to primary sulci. Extraction of secondary or tertiary sulci is increasingly unreliable due to variability in sulcal bifurcation, fragmentation, and absence, preventing the parcellation-based partitioning of sulci in the same way as other morphometric indices like cortical thickness or gray matter. Local gyrification index (LGI) is the ratio of cortical area within the sulcal folds to cortical area visible on the outer surface. Since LGI is reduced both by having fewer and shorter sulci, it is a direct reflection of sulcal length ^17, 18^. Moreover, LGI is the most sensitive and effective index of cortical folding to distinguish alterations in cortical folding, outperforming 22 curvature-based measures in identifying preterm from term infants ^17^. To overcome unreliability in whole-brain sulcal extraction, we use the LGI as a proxy for the deviations in sulcal length and shape that we detected in the PCS and STS between schizophrenia patients with and without hallucinations. The right and left hemisphere HCPMMP annotation files matching fsaverage were transformed to the individual cortical surface of each participant and the average LGI for each of the 360 parcellated brain regions (180 per hemisphere) included in the HCPMMP were extracted. The grand-average sulcal maps across all participants for the PCS and STS were thresholded at 25% of the image intensity ^19^ and projected on to fsaverage space using FSL and Freesurfer command tools. The HCP regions that corresponded to the PCS and STS were determined by visualizing the HCP labels atop the thresholded sulcal maps in fsaverage space in Freeview. The average LGI in the HCP parcels corresponding to the PCS and STS were respectively calculated for each hemisphere and correlated with the corresponding manually traced lengths. Pearson’s correlations were used to test the relationship between sulcal length and the respective average LGI in regions overlapping the PCS (HCP areas: 24dd, 24dv, SCEF, p32pr, a32pr, d32, p32, 8BM, 9m) and STS (HCP areas: STSdp, STSda, STSvp, STSva, A5, TPOJ1). Results are shown in Supplementary Figure S5.

**Atlas definition**

To interrogate the locus of putative sulcal shifts, we used the HCP-MMP1.0 multimodal surface-based anatomical by ^20^, which decomposes the cortex into 180 anatomical regions per hemisphere. The HCP parcellation was chosen for its finer-grained division of the medial prefrontal cortex in comparison to other atlases. Annotation files for the HCPMMP matching the standard stereotaxic brain (i.e fsaverage; Freesurfer v 6.0) were downloaded from <https://figshare.com/articles/HCP-MMP1_0_projected_on_fsaverage/3498446> and overlaid atop the fsaverage brain along with group-average sulcal maps for the PCS and STS. Cortical Explorer ([http://corticalexplorer.com](http://corticalexplorer.com/)), an interactive web-based user interface for parsing the HCP per-parcel data within a 3D scene, was used to visualize the different cortical areas ^21^.

**Lateralization of structural covariance networks for local gyrification index**

While common resting state networks are often operationalized as symmetric across hemispheres, recent work has evidenced hemispheric asymmetry in resting-state functional connectivity ^22, 23^, and this functional asymmetry may be reduced in schizophrenia patients ^24^. To investigate whether the lateralization of our sulcal length/depth findings were reflected in whole-brain intrinsic networks, we decomposed the 8 resting-state networks into their left and right hemisphere component brain regions. We averaged the LGI values between regions located within the same network, resulting in a 16x16 matrix for each group (Supplementary Figure S5). We again we performed nonparametric permutation testing with 5000 repetitions to test the statistical significance between structural covariance networks and corrected the resultant p-values by FDR < 0.05 (see Statistical analysis). We found that the mean LGI was significantly increased in patients with hallucinations compared to those without for the intra-hemispheric right salience network (R – R salience), the inter-hemisphere salience network (R – L salience), intra-left auditory network (L-L auditory), and between intra and inter salience and auditory networks (L salience – L auditory, R salience – L auditory, R salience – R auditory, L salience – R auditory) (Supplementary Table S7). These results suggest that hallucination status has a lateralized influence on the coordination of local gyrification within and between salience and auditory intrinsic networks.

**Imaging pre-processing and voxel-based morphometry analysis**

Structural MRI data were analyzed with FSL-VBM version 5.0.10 (<http://fsl.fmrib.ox.ac.uk/fsl>), an optimized VBM protocol ^25, 26^ carried out with FSL tools ^27^. First, all images were visually inspected to ensure no gross morphological abnormalities and were reoriented to match the orientation of the MNI152 template image. Quality of the structural data was quantitatively assessed with Freesurfer’s Euler number, an index of the topological complexity of the reconstructed cortical surface and reliable indicator of structural image quality ^28^. A 5-group 1-way ANOVA revealed no significant differences in the Euler number for the left hemisphere F(4,239)= 0.776, p=0.541, nor the right F(4,239)=1.402, p=0.234. Structural images were brain-extracted with FSL Brain Extraction Tool ^29^ and skull-stripped images were segmented into GM, WM, or CSF using FSL FAST. A study-specific template was created from n = 45 non-hallucinating subjects and a randomly selected subset of n = 45 hallucinating subjects, followed by non-linear registration to the ICBM-152 template, concatenation, and averaging of the resulting images. Images were smoothed using an isotropic Gaussian kernel with a sigma of 3.5 mm (FWHM ≅ 8 mm). To address the hypothesis of the GM changes in the paracingulate and medial prefrontal cortex (mPFC) regions, an 8mm region of interest (ROI) sphere was centered on coordinates sensitive to reality monitoring manipulations and hallucinations in schizophrenia ^6^. See Supplementary Table S6 for results.

**Supplementary Figures and Tables**


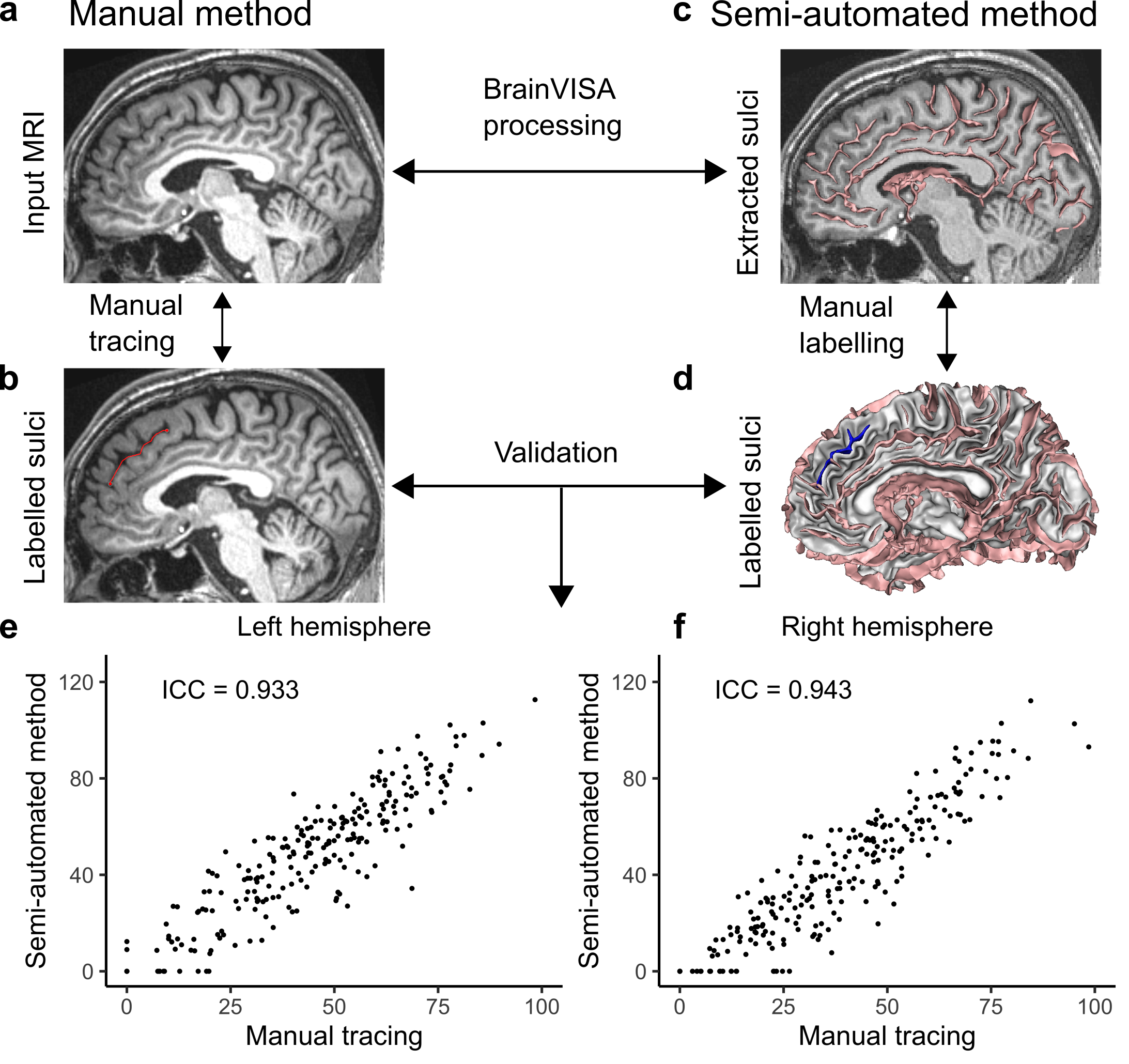


**Supplementary Figure S1. Validation of independent manual and semi-automated methods for paracingulate sulcus identification and length measurement. a–b.** The PCS was manually measured from T1 images using Multi-image Analysis GUI (Mango) medical imaging visualization software. **c.** Automated sulcal segmentation was performed using BrainVISA version 4.5 software. **d.** The PCS was manually labelled from BrainVISA whole-brain sulcal graphs. **e–f.** PCS lengths calculated from manual tracing and from BrainVISA processing were compared using the intraclass correlation coefficient (ICC).

**Supplementary Table S1.** Eligibility criteria for participants in UK multicentre and Shanghai studies.

|  | **UK, multi-centre** | **Shanghai, China** |
| --- | --- | --- |
| Inclusion criteria | - Meeting DSM-IV criteria for schizophrenia, schizophreniform, or schizoaffective psychosis as assessed by the research team - Within 5 years of first diagnosis - Male or female aged 16–35 years - IQ greater than 70 as assessed by the Wechsler Test of Adult Reading (WTAR) - Able to understand and give written informed consent - Participants were required to be taking stable antipsychotic treatment from a mental health-care team - Fluent in English | - Meeting DSM-IV criteria for schizophrenia using the Structured Clinical Interview for DSM-IV Axis I Disorders (SCID-I) - Male or female aged 16–50 years - Able to understand and give written informed consent |
| Exclusion criteria | - Current diagnosis of substance misuse - Current serious risk of suicide or violence - Had a relevant current or past medical disorder or were pregnant or breastfeeding - Used tetracycline antibiotics within 2 months of baseline visit or had a history of sensitivity or intolerance to an antibiotic - Meeting MRI exclusion criteria as defined by local scanning centre | - Current co-morbid DSM-IV Axis I disorder - History of other neurological, mental or substance disorder - History of receiving electroconvulsive therapy in the past six months - Meeting MRI exclusion criteria as defined by local scanning centre |

**Supplementary Table S2.** Reproducibility and comparability of scanning sequences for UK multicentre and Shanghai studies.

|  | **UK** | | | **China** |
| --- | --- | --- | --- | --- |
|  | **Manchester** | **Cambridge** | **Edinburgh** | **Shanghai** |
| MRI scanner | Philips Achieva | Siemens Trim Trio | Siemens Verio | Siemens |
| Field strength (Tesla) | 3 T | 3 T | 3 T | 3 T |
| Head coil | 8-channel | 12-channel | 12-channel | 32-channel |
| TE T1 (ms) | 3.1 | 2.98 | 2.98 | 3 |
| FOV T1 (mm) | 256 | 256 | 256 | 256 |
| Flip angle (deg) | 8 | 9 | 9 | 9 |
| Image matrix | 256 x 256 | 256 x 256 | 256 x 256 | 256 x 256 |
| Voxel dimensions (mm^3^) | 1 x 1 x 1.2 | 1 x 1 x 1 | 1 x 1 x 1.2 | 1 x 1 x 1 |
| Number of slices | 170 | 176 | 160 | 176 |

**Supplementary Table S3.** Intraclass correlation coeffecients (ICC) assessing consistency of PCS length measurements between raters. The PCS was measured for N=122 T1 scans among N=4 raters trained de novo on the manual measurement protocol, such that each scan was traced by at least 2 raters. Confidence intervals (CI) are reported for each ICC.

|  | **n observations** | **ICC** | **CI lower bound** | **CI upper bound** |
| --- | --- | --- | --- | --- |
| RH All | 122 | 0.968 | 0.965 | 0.972 |
| R1 vs. R2 | 30 | 0.971 | 0.962 | 0.977 |
| R1 vs. R3 | 31 | 0.978 | 0.972 | 0.983 |
| R1 vs. R4 | 15 | 0.964 | 0.949 | 0.975 |
| R3 vs. R4 | 24 | 0.946 | 0.914 | 0.961 |
| LH All | 122 | 0.935 | 0.929 | 0.942 |
| R1 vs. R2 | 30 | 0.959 | 0.947 | 0.968 |
| R1 vs. R3 | 28 | 0.934 | 0.914 | 0.949 |
| R1 vs. R4 | 11 | 0.931 | 0.893 | 0.956 |
| R3 vs. R4 | 25 | 0.917 | 0.836 | 0.942 |

**Supplementary Table S4.** Intraclass correlation coefficients (ICC) assessing reliability of paracingulate sulcus length measurements between manual, semi-automated (BrainVISA) methods.

|  | **n observations** | **ICC** | **CI lower bound** | **CI upper bound** |
| --- | --- | --- | --- | --- |
| RH Manual vs. Semi-automated | 237 | 0.943 | 0.938 | 0.948 |
| LH Manual vs. Semi-automated | 237 | 0.933 | 0.922 | 0.941 |

*BrainVISA sulcal extractions failed for N=7 scans.

**
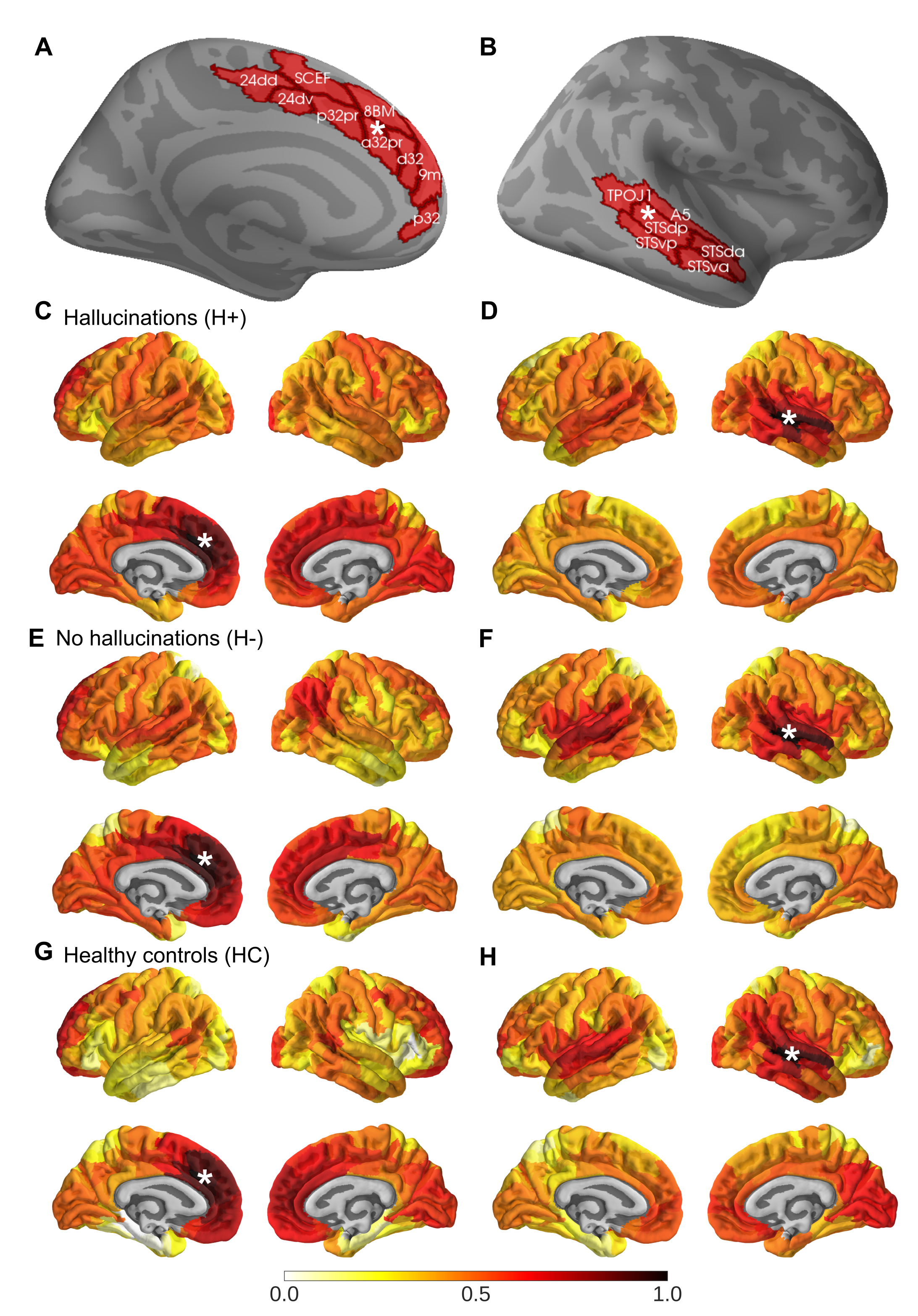
**

**Supplementary Figure S2.** **Whole-brain correlations for local gyrification index (LGI) in HCPMMP1.0-defined regions left a32pr and right STSdp.** A. HCP regions that overlap with the paracingulate sulcus (PCS) (24dd, 24dv, SCEF, p32pr, a32pr, d32, p32, 8BM, 9m). B. HCP regions that overlap with the superior temporal sulcus (STS) (STSdp, STSda, STSvp, STSva, A5, TPOJ1). A white asterisk indicates the seed regions left a32pr and right STSdp, which were the loci of the sulcal displacements identified for the PCS and STS, respectively. C–H. Correlation between LGI in seed regions and all other 360 regions defined by the HCPMMP1.0 atlas for C–D. Hallucinations (H+; n = 101); E–F. No hallucinations (H-; n = 80); G–H. Healthy controls (HC; n = 63). The colour scale represents the correlation coefficient (Pearson’s *r*) of each regional LGI measure for the HCP parcellation to the LGI in left a32pr (C, E, H) and right STSdp (D, F, G).


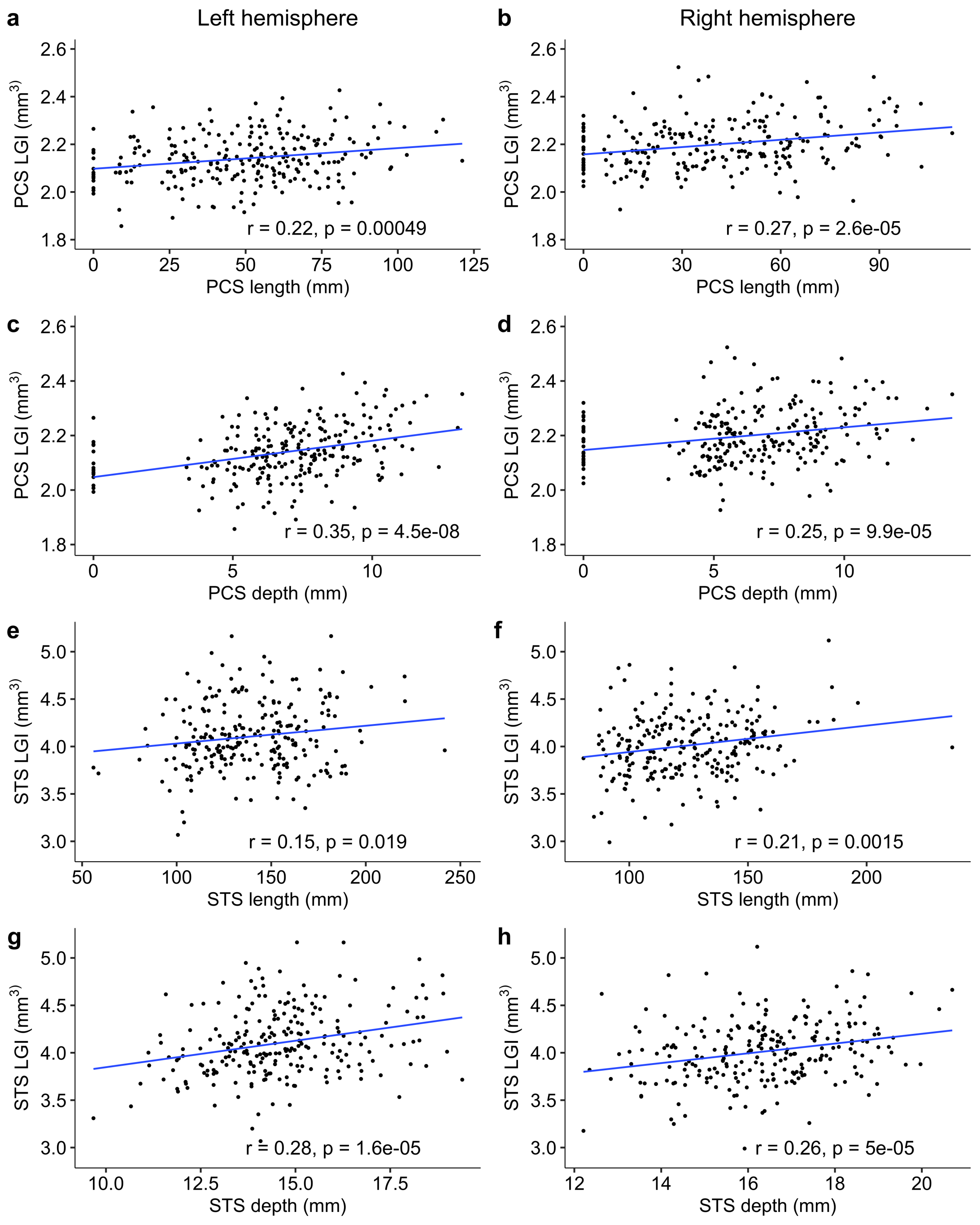


**Supplementary Figure S3.** Average local gyrification index (LGI) in regions corresponding to the paracingulate (PCS; HCP areas: 24dd, 24dv, SCEF, p32pr, a32pr, d32, p32, 8BM, 9m) and superior temporal sulci (STS; HCP areas: STSdp, STSda, STSvp, STSva, A5, TOPJ1) correlated against respective sulcal lengths (PCS: a–b, STS: e–f) and depths (PCS: c–d, STS: g–h) calculated from BrainVISA sulcal extractions for the left and right hemispheres, respectively.


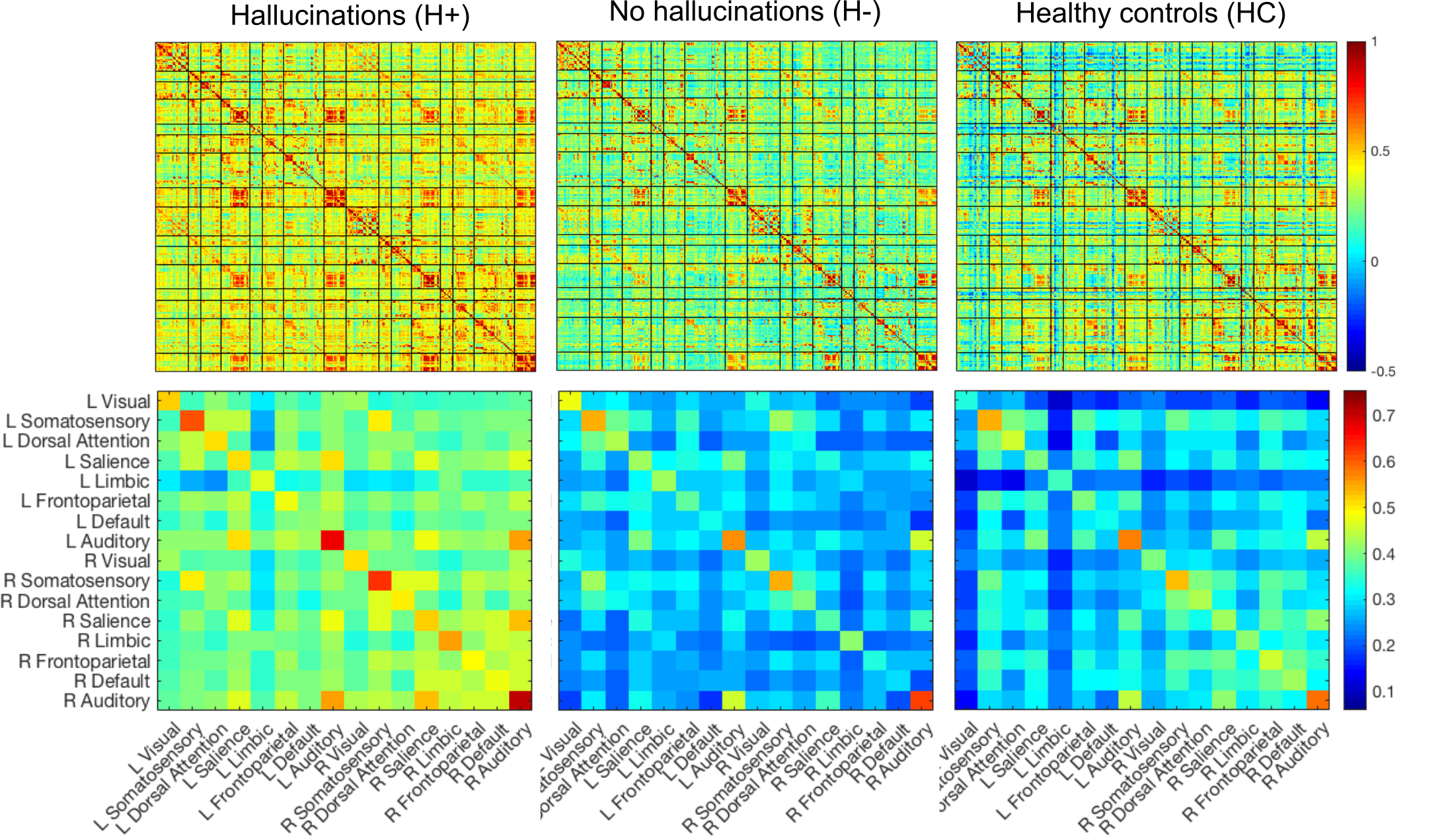


**Supplementary Figure S4. Lateralized resting-state networks.** Local gyrification index covariance matrices were re-ordered according the hemispheric (left, right) decomposition of 8 well-established and replicable resting-state networks. The LGI values between regions located within the same network were averaged, resulting in 16x16 group-wise matrices.


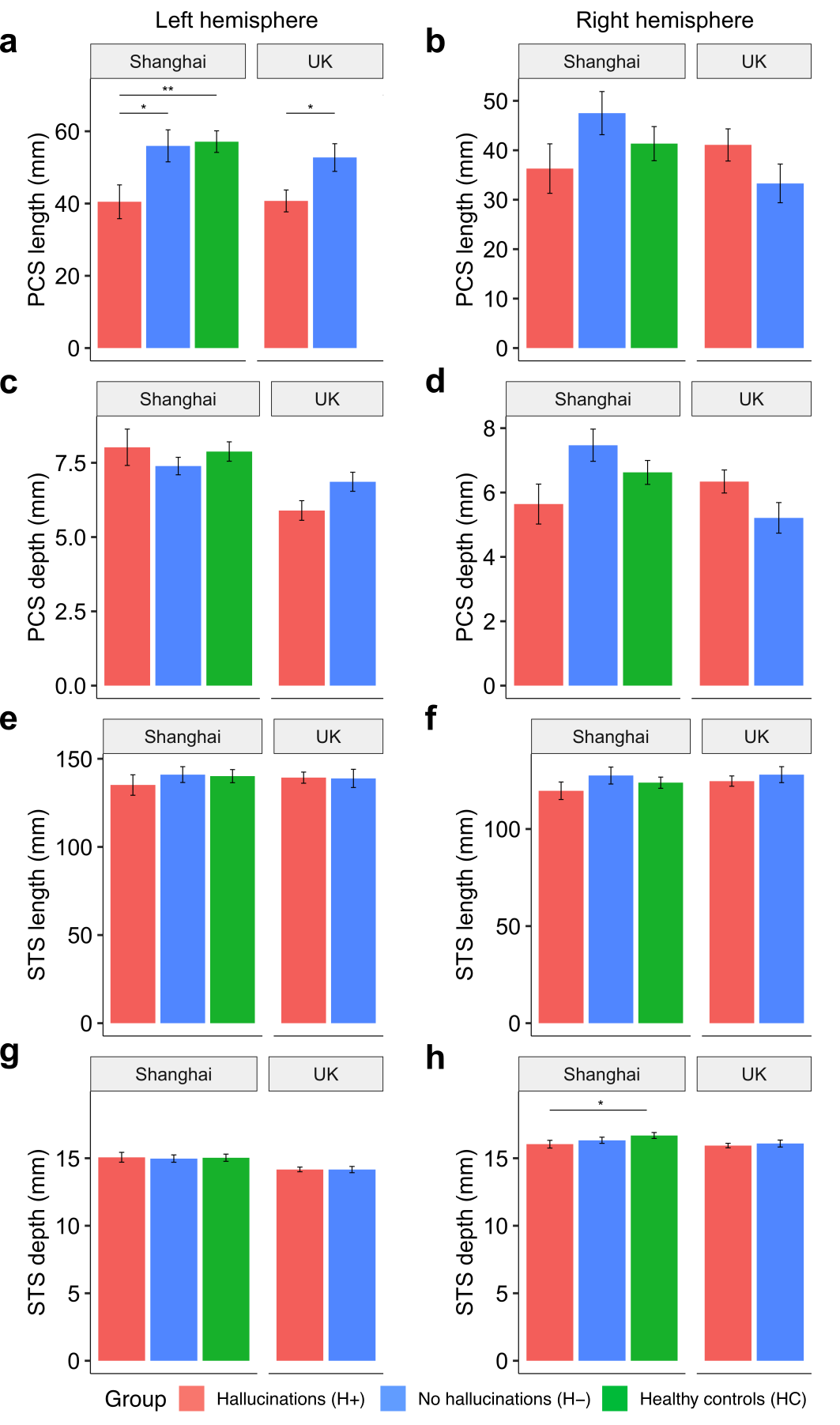


**Supplementary Figure S5.** Bilateral sulcal measurements displayed by hemisphere (left, right), group (H+, H-, HC) and dataset (Shanghai, UK) for the paracingulate sulcus (PCS) (length: **a–b**; depth: **c–d**) and superior temporal sulcus (STS) (length: **e–f**; depth: **g­–h**). Error bars denote the standard error of the mean (S.E.M.). * p<0.05; ** p<0.01. Numerical values (in mm) for the group means and S.E.M. (in parentheses) illustrated above:

**a)** Left hemisphere (LH) PCS length. Shanghai: H+ = 40.48 (4.68); H– = 55.96 (4.43); HC = 57.13 (2.99). UK: H+ = 40.72 (3.03); H– = 52.73 (3.83).

**b)** Right hemisphere (RH) PCS length. Shanghai: H+ = 36.30 (5.00); H– = 47.51 (4.37); HC = 41.33 (3.45). UK: H+ + 41.07 (3.25); H– = 33.30 (3.91).

**c)** LH PCS depth. Shanghai: H+ = 8.02 (0.61); H– = 7.39 (0.29); HC = 7.88 (0.32). UK: H+ = 5.89 (0.33); H– = 6.86 (0.32).

**d)** RH PCS depth. Shanghai: H+ = 5.64 (0.62); H– = 7.47 (0.50); HC = 6.62 (0.37). UK: H+ = 6.34 (0.36); H– = 5.21 (0.48).

**e)** LH STS length. Shanghai: H+ = 135.10 (5.80); H– = 141.03 (3.73); HC = 140.14 (4.49). UK: H+ = 139.29 (3.16); H– = 138.87 (5.16).

**f)** RH STS length. Shanghai: H+ = 119.71 (4.51); H– = 127.54 (4.37); HC = 123.85 (2.88). UK: H+ = 124.68 (2.65); H– = 128.00 (4.11).

**g)** LH STS depth. Shanghai: H+ = 15.0 (0.36); H– = 15.04 (0.27); HC = 14.97 (0.27). UK: H+ = 14.17 (0.17); H– = 14.28 (0.26).

**h)** RH STS depth. Shanghai: H+ = 16.04 (0.29); H– = 16.59 (0.26); HC = 16.87 (0.23). UK: H+ = 15.98 (0.17); H– = 16.24 (0.27).

**Supplementary Table S5.** Correspondence between HCPMMP1.0 areas and large-scale network.

| **Network** | **HCP area name** |
| --- | --- |
| Visual | V1 |
|  | MST |
|  | V6 |
|  | V2 |
|  | V3 |
|  | V4 |
|  | V8 |
|  | V3A |
|  | V7 |
|  | IPS1 |
|  | FFC |
|  | V3B |
|  | LO1 |
|  | LO2 |
|  | PIT |
|  | MT |
|  | ProS |
|  | PHA1 |
|  | PHA3 |
|  | PH |
|  | DVT |
|  | V6A |
|  | VMV1 |
|  | VMV3 |
|  | PHA2 |
|  | V4t |
|  | FST |
|  | V3CD |
|  | LO3 |
|  | VMV2 |
|  | VVC |
| Somatosensory | 4 |
|  | 3b |
|  | 5m |
|  | 5L |
|  | 24dd |
|  | 24dv |
|  | 1 |
|  | 2 |
|  | 3a |
|  | 6d |
|  | 6mp |
|  | 6v |
| Dorsal attention | PEF |
|  | 7AL |
|  | 7Am |
|  | 7PL |
|  | 7PC |
|  | LIPv |
|  | VIP |
|  | MIP |
|  | IFJp |
|  | LIPd |
|  | 6a |
|  | PFt |
|  | AIP |
|  | TE2p |
|  | PHT |
|  | TPOJ2 |
|  | TPOJ3 |
|  | PGp |
|  | IP0 |
| Salience | FEF |
|  | 5mv |
|  | 23c |
|  | SCEF |
|  | 6ma |
|  | p24pr |
|  | 33pr |
|  | a24pr |
|  | p32pr |
|  | 6r |
|  | 46 |
|  | 9-46d |
|  | 43 |
|  | PFcm |
|  | PoI2 |
|  | FOP4 |
|  | MI |
|  | AVI |
|  | AAIC |
|  | FOP1 |
|  | FOP3 |
|  | FOP2 |
|  | PFop |
|  | PF |
|  | PoI1 |
|  | FOP5 |
|  | a32pr |
| Limbic | 10v |
|  | 10pp |
|  | 13l |
|  | OFC |
|  | EC |
|  | PeEc |
|  | TGd |
|  | TF |
|  | 25 |
|  | pOFC |
|  | TGv |
|  | PI |
| Frontoparietal | 55b |
|  | POS2 |
|  | 7Pm |
|  | 8BM |
|  | 8C |
|  | 44 |
|  | a47r |
|  | IFJa |
|  | IFSp |
|  | IFSa |
|  | p9-46v |
|  | a9-46v |
|  | a10p |
|  | 11l |
|  | i6-8 |
|  | TE1p |
|  | IP2 |
|  | IP1 |
|  | PFm |
|  | p47r |
| Default mode | RSC |
|  | SFL |
|  | PCV |
|  | 7m |
|  | POS1 |
|  | 23d |
|  | v23ab |
|  | d23ab |
|  | 31pv |
|  | a24 |
|  | d32 |
|  | p32 |
|  | 10r |
|  | 47m |
|  | 8Av |
|  | 8Ad |
|  | 9m |
|  | 8BL |
|  | 9p |
|  | 10d |
|  | 45 |
|  | 47l |
|  | 9a |
|  | 47s |
|  | s6-8 |
|  | Pir |
|  | PreS |
|  | H |
|  | TE1a |
|  | TE2a |
|  | PGi |
|  | PGs |
|  | 31pd |
|  | 31a |
|  | s32 |
|  | p10p |
|  | TE1m |
|  | p24 |
| Auditory | A1 |
|  | PSL |
|  | STV |
|  | OP4 |
|  | OP1 |
|  | OP2-3 |
|  | 52 |
|  | RI |
|  | TA2 |
|  | STGa |
|  | PBelt |
|  | A5 |
|  | STSda |
|  | STSdp |
|  | STSvp |
|  | TPOJ1 |
|  | Ig |
|  | MBelt |
|  | LBelt |
|  | A4 |
|  | STSva |

**Supplementary Table S6.** Group-wise asymmetry indices and measurements of sulcal length and depth for paracingulate and superior temporal sulci.

|  | | | **H+ (N=101)** | **H- (N=80)** | **HC (N=63)** | **Test statistic** | **q-value** |
| --- | --- | --- | --- | --- | --- | --- | --- |
| Para-cingulate sulcus (PCS) | Length | L, mm (SE) | 40.67 (2.58) | 54.24 (2.89) | 57.14 (2.99) |  |  |
|  |  | R, mm (SE) | 40.06 (2.77) | 39.95 (3.01) | 41.33 (3.45) |  |  |
|  |  | AI, % (SE) | –3.92 (10.65) | –41.08 (10.04) † | –39.17 (10.54) † | F(2,229)=4.25 | 0.0404* |
|  | Depth | L, mm (SE) | 6.33 (0.302) | 7.11 (0.220) | 7.88 (0.326) |  |  |
|  |  | R, mm (SE) | 6.19 (0.311) | 6.27 (0.365) | 6.62 (0.372) |  |  |
|  |  | AI, % (SE) | –4.13 (9.46) | –26.72 (8.06) † | –20.76 (8.83) † | F(2,227)=1.87 | 0.275 |
| Superior temporal sulcus (STS) | Length | L, mm (SE) | 138.41 (2.77) | 139.88 (3.44) | 140.15 (3.73) |  |  |
|  |  | R, mm (SE) | 123.63 (2.30) | 127.79 (2.97) | 123.85 (2.88) |  |  |
|  |  | AI, % (SE) | –10.84 (2.67) † | –8.41 (3.30) † | –11.64 (3.50) † | F(2,234)=0.272 | 0.841 |
|  | Depth | L, mm (SE) | 14.36 (0.160) | 14.61 (0.190) | 15.04 (0.267) |  |  |
|  |  | R, mm (SE) | 15.99 (0.146) | 16.41 (0.186) | 16.87 (0.233) |  |  |
|  |  | AI, % (SE) | +10.93 (1.21) † | +11.69 (1.28) † | +11.76 (1.61) † | F(2,234)=0.129 | 0.879 |

Positive values represent longer or deeper sulci in the right hemisphere. UK and Shanghai samples were merged due to ethnic invariance on sulcal morphology.

† FDR<0.05 for sulcal AI assessed within each group using 1-sample t-test.

* FDR<0.05 for sulcal AI assessed between groups using 1-way ANOVA.

*BrainVISA sulcal extractions failed for N=7 scans. Computational errors in calculating sulcal metrics occurred for N=6 scans for PCS length and N=7 scans for PCS depth.

**Supplementary Table S7.** Mean local gyrification covariance within and between lateralized RSNs for the auditory and salience networks.

|  | Hallucinations (H+) | No hallucinations (H-) | Health controls (HC) | q-value |
| --- | --- | --- | --- | --- |
| L Salience – L Salience | 0.5123 (0.4851–0.541) | 0.4277 (0.4006–0.4557) | 0.4061 (0.3811–0.4316) | 0.0566 |
| R Salience – R Salience | 0.5195 (0.4934–0.5454) | 0.4581 (0.3374–0.3922) | 0.4267 (0.4018–0.4493) | 0.0120 |
| L Salience – R Salience | 0.4651 (0.4427–0.4904) | 0.3337 (0.3156–0.3558) | 0.3386 (0.3193–0.3615) | 0.0131 |
| L Auditory – L Auditory | 0.6773 (0.6272–0.714) | 0.5629 (0.5118–0.6062) | 0.5764 (0.5151–0.6206) | 0.0131 |
| R Auditory – R Auditory | 0.7053 (0.6538–0.7391) | 0.6193 (0.5679–0.6606) | 0.5917 (0.523–0.6311) | 0.0566 |
| L Auditory – R Auditory | 0.5495 (0.5117–0.5788) | 0.4525 (0.4037–0.4919) | 0.4506 (0.4214–0.4763) | 0.0566 |
| L Salience – L Auditory | 0.5161 (0.4778–0.5508) | 0.3971 (0.3572–0.4337) | 0.3989 (0.359–0.433) | 0.0131 |
| R Salience – R Auditory | 0.5299 (0.4698–0.5871) | 0.3544 (0.2767–0.4343) | 0.4089 (0.3522–0.4668) | 0.004 |
| L Salience – R Auditory | 0.4723 (0.431–0.5107) | 0.324 (0.2755–0.3804) | 0.3142 (0.2627–0.3661) | 0.0131 |
| R Salience – L Auditory | 0.4753 (0.4366–0.5134) | 0.3214 (0.2877–0.358) | 0.3783 (0.3429–0.4171) | 0.0131 |

95% C.I. are reported in brackets.

**
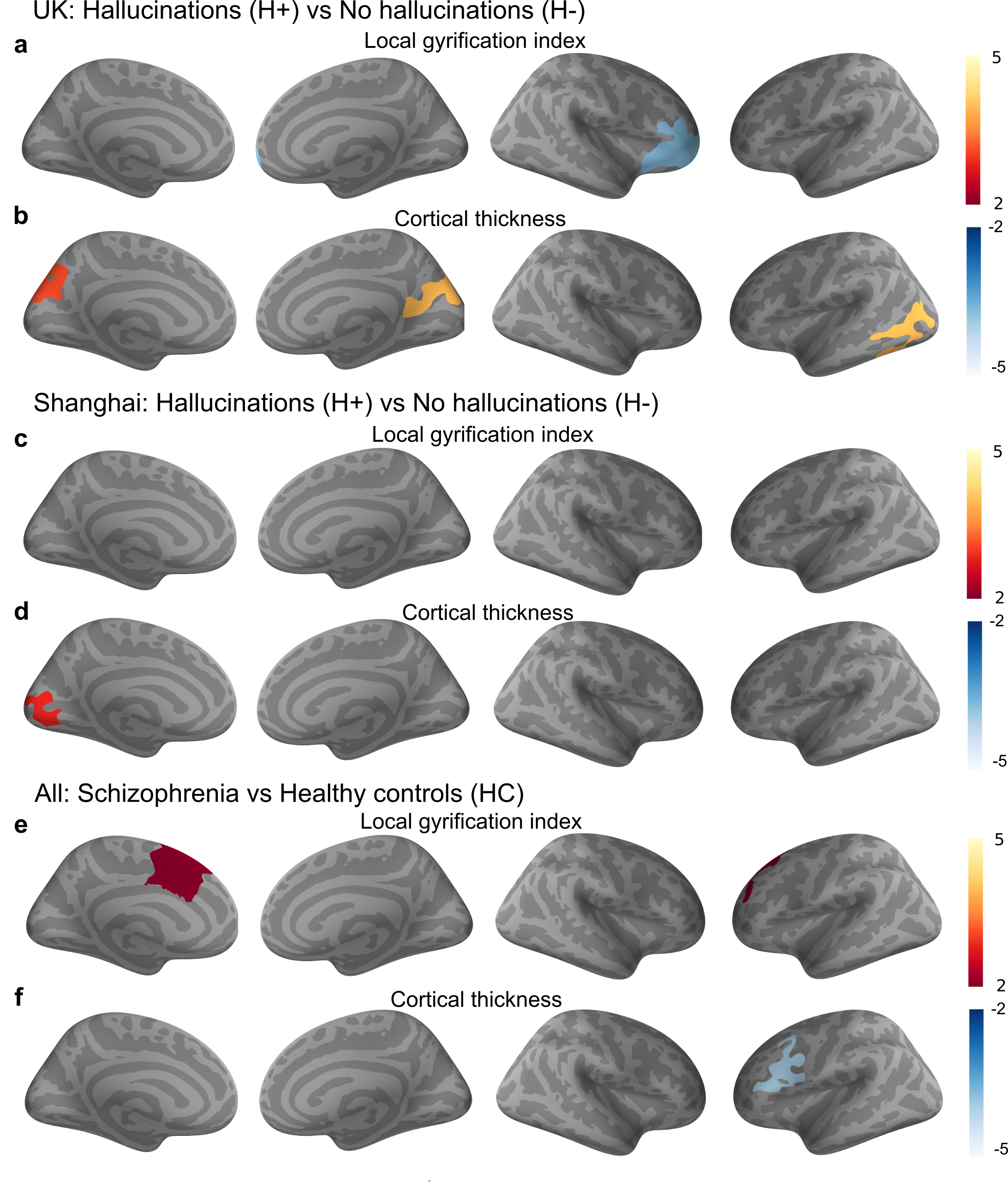
**

**Supplementary Figure S6.** Cluster showing significant difference in the local gyrification index and cortical thickness between schizophrenia patients with hallucinations (H+) and those without hallucinations (H-) for the UK **(a­–b)** and Shanghai **(c­–d)** datasets, as well as between schizophrenia patients (H+ and H- combined) compared to healthy controls for the Shanghai dataset **(e–f)**. Cortical statistical maps displaying decreased gyrification in H+ in the rostral middle frontal cortex for the UK sample, increased thickness in H+ for left lingual gyrus for both datasets and left inferior parietal and bilateral precuneus for the UK dataset. Schizophrenia patients show increased gyrification in the left superior frontal cortex and reduced thickness in the left pars opercularis compared to healthy controls. p<0.05, FDR-corrected for multiple comparisons. All results were corrected for multiple comparisons using Monte Carlo simulation with 10 000 iterations with a cluster-forming threshold of p<0.05 and corrected for multiple comparisons across each hemisphere. The colour bar shows the logarithmic scale of p values (−log10).

**Supplementary Table S8.** Results from voxel-based morphometry analysis to identify clusters in which grey matter volume differed between schizophrenia patients with and without hallucinations within a region of interest (ROI) in the medial prefrontal cortex (mPFC).

|  | **UK, multi-centre** | | | | | | **Shanghai, China** |
| --- | --- | --- | --- | --- | --- | --- | --- |
|  | TFCE p-value | cluster size | MNI coordinates | | | t-value | TFCE p-value |
|  |  |  | x | y | z |  |  |
| Hallucinations > No hallucinations | 0.0012 | 149 | 0 | 56 | 4 | 7.87 | 0.322 |

Nonparametric 2-sample t-tests were performed using FSL randomise with 5000 permutations and using threshold free cluster enhancement (TFCE) to identify clusters in which grey matter volume differed between groups at a statistical threshold of p<0.05 within the ROI. Age and sex were included as confound regressors for the Shanghai data, and age, sex, and scanner for the UK data. GM volume was significantly greater in schizophrenia patients with hallucinations compared to those without in the mFPC ROI for the UK dataset, as has been found previously (Garrison et al., 2015), but was not significant for the Shanghai dataset.

**Supplementary references**

1. Deakin B *et al.* The benefit of minocycline on negative symptoms of schizophrenia in patients with recent-onset psychosis (BeneMin): a randomised, double-blind, placebo-controlled trial. *Lancet Psychiatry* 2018; **5**(11)**:** 885-894.

2. Li Z *et al.* Striatal dysfunction in patients with schizophrenia and their unaffected first-degree relatives. *Schizophr Res* 2018; **195:** 215-221.

3. van Tol MJ *et al*. Voxel-based gray and white matter morphometry correlates of hallucinations in schizophrenia: The superior temporal gyrus does not stand alone. *Neuroimage Clin* 2014; **4:** 249-257.

4. Kim SH *et al.* The usefulness of a self-report questionnaire measuring auditory verbal hallucinations. *Prog Neuropsychopharmacol Biol Psychiatry* 2010; **34**(6)**:** 968-973.

5. Steel C *et al.* The multidimensional measurement of the positive symptoms of psychosis. *Int J Methods Psychiatr Res* 2007; **16**(2)**:** 88-96.

6. Garrison JR *et al*. Paracingulate sulcus morphology is associated with hallucinations in the human brain. *Nat Commun* 2015; **6:** 8956.

7. Mangin JF *et al.* A framework to study the cortical folding patterns. *Neuroimage* 2004; **23 Suppl 1:** S129-138.

8. Ochiai T *et al.* Sulcal pattern and morphology of the superior temporal sulcus. *Neuroimage* 2004; **22**(2)**:** 706-719.

9. Wei X *et al.* Paracingulate Sulcus Asymmetry in the Human Brain: Effects of Sex, Handedness, and Race. *Sci Rep* 2017; **7:** 42033.

10. Dale AM, Fischl B, Sereno MI. Cortical surface-based analysis. I. Segmentation and surface reconstruction. *Neuroimage* 1999; **9**(2)**:** 179-194.

11. Fischl B, Dale AM. Measuring the thickness of the human cerebral cortex from magnetic resonance images. *Proc Natl Acad Sci U S A* 2000; **97**(20)**:** 11050-11055.

12. Fischl B, Liu A, Dale AM. Automated manifold surgery: constructing geometrically accurate and topologically correct models of the human cerebral cortex. *IEEE Trans Med Imaging* 2001; **20**(1)**:** 70-80.

13. Fischl B *et al.* Whole brain segmentation: automated labeling of neuroanatomical structures in the human brain. *Neuron* 2002; **33**(3)**:** 341-355.

14. Han X *et al.* Reliability of MRI-derived measurements of human cerebral cortical thickness: the effects of field strength, scanner upgrade and manufacturer. *Neuroimage* 2006; **32**(1)**:** 180-194.

15. Schaer M *et al*. A surface-based approach to quantify local cortical gyrification. *IEEE Trans Med Imaging* 2008; **27**(2)**:** 161-170.

16. Schaer M *et al*. How to measure cortical folding from MR images: a step-by-step tutorial to compute local gyrification index. *J Vis Exp* 2012; (59)**:** e3417.

17. Shimony JS *et al.* Comparison of cortical folding measures for evaluation of developing human brain. *Neuroimage* 2016; **125:** 780-790.

18. Kochunov P, Rogers W, Mangin JF, Lancaster J. A library of cortical morphology analysis tools to study development, aging and genetics of cerebral cortex. *Neuroinformatics* 2012; **10**(1)**:** 81-96.

19. Jahn A, Nee DE, Alexander WH, Brown JW. Distinct Regions within Medial Prefrontal Cortex Process Pain and Cognition. *J Neurosci* 2016; **36**(49)**:** 12385-12392.

20. Glasser MF *et al.* A multi-modal parcellation of human cerebral cortex. *Nature* 2016; **536**(7615)**:** 171-178.

21. Budd S. The Cortical Explorer: A web-based user-interface for exploration of brain data. MEng Computing thesis, Imperial College of Science, Technology and Medicine, London, 2017.

22. Raemaekers M, Schellekens W, Petridou N, Ramsey NF. Knowing left from right: asymmetric functional connectivity during resting state. *Brain Struct Funct* 2018; **223**(4)**:** 1909-1922.

23. Karolis VR, Corbetta M, Thiebaut de Schotten M. The architecture of functional lateralisation and its relationship to callosal connectivity in the human brain. *Nat Commun* 2019; **10**(1)**:** 1417.

24. Agcaoglu O *et al.* Decreased hemispheric connectivity and decreased intra- and inter- hemisphere asymmetry of resting state functional network connectivity in schizophrenia. *Brain Imaging Behav* 2018; **12**(3)**:** 615-630.

25. Ashburner J, Friston KJ. Voxel-based morphometry--the methods. *Neuroimage* 2000; **11**(6 Pt 1)**:** 805-821.

26. Good CD *et al*. A voxel-based morphometric study of ageing in 465 normal adult human brains. *Neuroimage* 2001; **14**(1 Pt 1)**:** 21-36.

27. Smith SM *et al.* Advances in functional and structural MR image analysis and implementation as FSL. *Neuroimage* 2004; **23 Suppl 1:** S208-219.

28. Rosen AFG *et al*. Quantitative assessment of structural image quality. *Neuroimage*. 2018;169:407-418. doi:10.1016/j.neuroimage.2017.12.059

29. Popescu V *et al.* Optimizing parameter choice for FSL-Brain Extraction Tool (BET) on 3D T1 images in multiple sclerosis. *Neuroimage* 2012; **61**(4)**:** 1484-1494.
